# Anæsthetics, and the Physiology of the Heart

**Published:** 1894-04

**Authors:** 


ARTICLE VI.

### ANÆSTHETICS, AND THE PHYSIOLOGY OF THE HEART.

At the regular meeting of the Odontological Society of Great Britain, last December, a very concise and scientific paper was read by John W. Pickering, D.Sc. (Lond.), on the "Physiology of the Heart in relation to Anæsthetics," treating the subject from an experimental standpoint. The author showed that the following are some of the possible modes of action on the heart:

(1) The anæsthetic acts on the cardiac muscle itself, and may directly paralyze its contractile power.

(2) That the paralysis of the heart when present, is due to the action of the anæsthetic of the intrinsic cardiac nervous mechanism.

(3) That chloroform syncope is due to a reflex cardiac inhibition caused by irritation of the nerve ending of the vagi in the lungs.

(4) That chloroform primarily paralyses the respiratory centre in the medulla oblongata, and that the consequent asphyxial condition of the blood secondarily paralyzes the heart.

(5) That cardiac dilation, when present, is due to pulmonary obstruction, and that chloroform has no specific action on the heart.

This last view, however, has been rendered improbable by recent experiments, which have shown that chloroform will produce dilation of *both sides* of the heart.

Dr. Pickering experimented on the hearts of embryos previous to the development of a functional nervous mechanism—the chick embryo between the fiftieth and eightieth hour of incubation presenting an accessible form of heart or nervous system. Though the embryonic circulation is then very active; there is no complication due to a change of blood-pressure; also the factor of asphyxia is eliminated, except when purposely introduced, by placing the embryo in an atmosphere of carbonic acid. The experiments were made on hearts *in situ*, and under conditions such that their rhythms were maintained for many hours unchanged, provided that no chemical or physical stimuli were applied to them. Chloroform injected under the blastoderm of the embryo, rapidly reduced its cardiac rhythm, and produced an exaggerated diastole. Either acted as a powerful stimulant to the embryonic heart. These experiments show that chloroform has a depressor and ether an augumentor action—a conclusion at variance with the view of Claude Bernard, reiterated by the Hyderabad Commission. Nitrous oxide and air has but little depressant effect on the embyronic heart; pure nitrous oxide stops the embryonic heart in diastole. Dr. Pickering showed that there is apparently less danger of cardiac stoppage with the use of oxygen and chloroform, than with chloroform alone.

As to the vexed question as to whether nitrous oxide forms a compound with any of the constituents of the blood, or whether its action is owing to the formation of reduced hæmoglobin and consequent deprivation of the tissues of oxygen, Dr. Dudley Buxton urged the probable formation of compound of nitrous oxide with hæmoglobin, or with some globulin of the plasma. Recent researches lend much probability to these views. The question, however, is left *sub judice.*

Is it possible pharmacologically to antagonize the depressant action of anæsthetics on the heart?

An application of a 1 per cent. solution of ammonium hydrate directly to the ventricle of a frog's heart, restores it almost to its original power. This is proved in experiments on the embryonic heart.

Dr. Wood, of Philadelphia, failed to get any restoration of rhythm by hypodermic injection of atropine, amyl nitrate, or caffeine, while alcohol increased the cardiac depression. Ammonia had slightly beneficial effects, and digitalis, by raising the blood pressure, often averted death. Strychnine, in doses of .00002 grain, increases both the force and frequency of the embryonic heart rhythm. Large doses are depressant. Chloroformed hearts will, if not too strongly poisoned, respond to electrical stimuli. The direct application of heat will often restore a chloroformed heart when chemical and electrical stimuli fail. Dr. Pickering closed by advising the trial of external heat in form of wet rags over the heart.

---

ARTICLE VII.

